# Ultra-Sensitive NT-proBNP Quantification for Early Detection of Risk Factors Leading to Heart Failure

**DOI:** 10.3390/s17092116

**Published:** 2017-09-14

**Authors:** Keum-Soo Song, Satish Balasaheb Nimse, Mukesh Digambar Sonawane, Shrikant Dashrath Warkad, Taisun Kim

**Affiliations:** Institute for Applied Chemistry and Department of Chemistry, Hallym University, Chuncheon 200-702, Korea; hanlimsk@empal.com (K.-S.S.); satish_nimse@hallym.ac.kr (S.B.N.); mukeshsonawane87@hallym.ac.kr (M.D.S.); shrikant.warkad@hallym.ac.kr (S.D.W.)

**Keywords:** NT-proBNP, heart failure, left ventricular hypertrophy, 9G DNAChip, DAGON, cardiovascular disease

## Abstract

Cardiovascular diseases such as acute myocardial infarction and heart failure accounted for the death of 17.5 million people (31% of all global deaths) in 2015. Monitoring the level of circulating N-terminal proBNP (NT-proBNP) is crucial for the detection of people at risk of heart failure. In this article, we describe a novel ultra-sensitive NT-proBNP test (*us*-NT-proBNP) that allows the quantification of circulating NT-proBNP in 30 min at 25 °C in the linear detection range of 7.0–600 pg/mL. It is a first report on the application of a fluorescence bead labeled detection antibody, DNA-guided detection method, and glass fiber membrane platform for the quantification of NT-proBNP in clinical samples. Limit of blank, limit of detection, and limit of quantification were 2.0 pg/mL, 3.7 pg/mL, and 7 pg/mL, respectively. The coefficient of variation was found to be less than 10% in the entire detection range of 7–600 pg/mL. The test demonstrated specificity for NT-proBNP without interferences from bilirubin, intra-lipid, biotin, and hemoglobin. The serial dilution test for plasma samples containing various NT-proBNP levels showed the linear decrement in concentration with the regression coefficient of 0.980–0.998. These results indicate that *us*-NT-proBNP test does not suffer from the interference of the plasma components for the measurement of NT-proBNP in clinical samples.

## 1. Introduction

The World Health Organization (WHO) factsheet reviewed in 2016 acknowledges that cardiovascular diseases (CVDs) are the number one cause of death worldwide. CVDs accounted for the death of 17.5 million people (31% of all global deaths) in 2015 [1,2], a number that is expected to surpass 23.6 million by 2030 [3]. Acute myocardial infarction (AMI) and heart failure (HF) are the most common CVDs [4,5,6]. A policy statement from the American Heart Association projected that the prevalence of HF would increase 46% from 2012 to 2030, resulting in >8 million people ≥18 years of age with HF in the United States [7]. HF affects about 25 million people globally [8,9].

HF is a progressive condition that begins with risk factors for left ventricular systolic dysfunction (LVSD), proceeds to asymptomatic changes in a cardiac structure and function such as left ventricular hypertrophy (LVH) and then evolves into a clinically overt HF, disability, and death [10].

Recent reports suggest that one in four middle-aged adults will develop heart failure if they survive to age 85 years. However, enabling people to reach middle age without cardiovascular risk factors can preserve their cardiac function and reduce their lifetime risk of heart failure [11]. Therefore, to reduce the burden of CVDs on the global healthcare programs by preserving the cardiac function, people who are at high cardiovascular risk need early detection, management using counseling, and medicines [12].

N-terminal proBNP (NT-proBNP) is released in response to myocyte stretching resulting from left ventricle hypertrophy [13]. NT-proBNP is a crucial biomarker for the detection of people at the risk of HF [14,15,16]. Hence, monitoring of NT-proBNP level recognizes individuals at a risk of adverse cardiovascular events such as LVH, LVSD, and HF [17,18,19]. The ability to identify patients at an increased risk of LVH, LVSD, and low ejection fraction in the early stage is a crucial step to manage and preserve the cardiovascular function [20,21,22]. Therefore, a test that detects NT-proBNP at an ultra-low concentration with high precision in a routine health care check-up setting is crucial for the early identification of conditions leading to HF.

Several analytical methods have been reported for the detection of circulating NT-proBNP in past decades, including radioimmunoassay [23,24], immunoradiometric assay [25], enzyme-linked immunosorbent assay (ELISA) [26] and electrochemiluminescence immunoassay (ECLIA) [27]. Conventional ELISA methods are time consuming and need more sample volume. Whereas, radioimmunoassay and immunoradiometric assay are prone to radionuclide pollution problems. A recently reported ECLIA was found to detect NT-proBNP in the linear detection range of 0.1 pg/mL to 25 ng/mL [28]. Even though ECLIA is known for high sensitivity and specificity, it requires high installation cost, large analytical instrument, well-trained personnel. An amperometric magneto-immunosensor using an indirect competitive format is recently reported for the detection NT-proBNP with the detection range of 120 pg/mL to 42.9 ng/mL [29]. Therefore, exploring a novel, simple, rapid, sensitive, and specific method for the quantification of NT-proBNP to assess the individuals risk to HF at an early stage and to improve the treatment success has secured considerable interest.

A recently reported DNA-guided detection (DAGON) method based on 9G DNAChip platform allows quantification of biomarkers [30]. In DAGON, a biomolecular complex of Cy5-labelled detection antibody, target antigen, and capture antibody-DNA conjugate is allowed to form in the solution. The biomolecular complex is allowed to hybridize with immobilized probes on the chip surface at the room temperature and detected. A recent application of DAGON on a microarray allowed the quantification of NT-proBNP in plasma with the LoD of 6.8 pg/mL, but required overnight incubation [31]. The LoD and the time of detection were limited by the use of Cy5-labeled secondary antibody. Thus, we proposed the labeling of biomolecules with fluorescent beads (FB) instead of Cy5 dye.

In this article, we describe a novel ultra-sensitive NT-proBNP (*us*-NT-proBNP) test that allows the quantification of circulating NT-proBNP in 30 min at 25 °C in the linear detection range of 7.0 pg/mL~600 pg/mL. *us*-NT-proBNP test is based on a glass fiber membrane platform (GFMP). It is a first report on the application of a FB labeled detection antibody, DAGON, and a GFMP for the quantification of NT-proBNP in clinical samples.

## 2. Materials and Methods

All chemicals were purchased from Sigma-Aldrich Chemicals (Yongin-si, South Korea). All the oligonucleotides were purchased from Bioneer (Daejeon, South Korea). A standard sample of recombinant NT-proBNP recombinant antigen (Catalog #. 8NT2) was obtained from the HyTest Ltd. (Turku, Finland). The monoclonal mouse anti-human NT-proBNP capture antibody (Catalog #. 8NT2-15F11) and detection antibody (Catalog #. 8NT2-24E11) were also purchased from the HyTest. Hemoglobin (Catalog #. H7379) and biotin (Catalog #. B4501) were purchased from Sigma-Aldrich (St. Louis, MO, USA). Carboxyl group-modified fluorescent beads of 0.2 µm size (excitation wavelength 622 nm and emission wavelength 645 nm, Catalog #. F8806) were purchased from Life Technologies Corporation (Eugene, OR, USA).

### 2.1. Clinical Samples

Plasma samples (*n* = 58) from individuals including men and women of different age groups were collected at Fuwai Hospital, Beijing, China, during 17 to 20 of January 2017. The research and ethics committee of Fuwai Hospital, Beijing, China approved the study. Clinical samples of individuals for whom NT-proBNP was requested as a part of routine health check-up were used. There were no exclusion criteria. The NT-proBNP concentrations were pre-determined by Elecsys^®^ NT-proBNP assay using the Roche Elecsys^®^ E601 analyzer (Roche Diagnostics, Changning, China), and these samples were used to evaluate the clinical performance of *us*-NT-proBNP test.

Blood was collected into the EDTA tubes and gently mixed by inverting ten times and refrigerated at 4 °C for less than 4 h. Plasma was removed by centrifugation at 1800 g for 10 min. Separated plasma samples were pipetted into the clean plastic screw-cap vials, and the vials were labelled. Care was taken to avoid transfer of red blood cells into the vials. Vial caps were screwed firmly to prevent leakage and stored at 4 °C. Plasma samples were kept in BMT Thermo Control (Biometrix Technology Inc., Chuncheon, South Korea) for 10 min at 25 °C before the test and then used immediately.

### 2.2. us-NT-proBNP Test

*us*-NT-proBNP test is performed on a lateral flow membrane test strip (LFMTS) containing GFMP. The GFMP in LFMTS contains test and control lines on which the oligonucleotide probes are immobilized using 9G technology [32,33]. Recently reported DNA-guided detection (DAGON) method was used in *us*-NT-proBNP test to measure NT-proBNP levels in clinical samples.

In DAGON method, a biomolecular complex of the fluorobead-labelled detection antibody (FB-dAB), the capture antibody–DNA conjugate (cAB-DNA), and the target antigen (NT-proBNP) is allowed to form in the solution. The labelling of detection and capture antibodies with FB and DNA respectively were done by following the reported method [34,35]. In brief, the carboxylic acid functional groups on the surface of FB were first activated by reacting them with the EDC (1-ethyl-3-(3-dimethylaminopropyl) carbodiimide hydrochloride) cross-linker. The activated FB were then allowed to react with the amine functions in dAB to produce FB-dAB conjugates. For the synthesis of cAB-DNA, the cAB was first activated by reacting them with 2-iminothiolane in bicarbonate buffer. The amine modified DNAs were activated with sulfo-SMCC (sulfosuccinimidyl 4-(*N*-maleimido-methyl)cyclohexane-1-carboxylate) linker in 1× PBS buffer to obtain the DNA-sulfo-SMCC. Then the iminothiolane-activated cAB was reacted with the DNA-sulfo-SMCC in 1× PBS buffer solution to obtain cAB-DNA. The Cy5-DNA were obtained by reacting the amine functions in the amine modified DNA with the Cy5 Dye mono-reactive NHS ester according to the standard protocol provided by the manufacturer with the mono-reactive Cy5DyeTM (GE Healthcare UK Ltd., Buckinghamshire, UK).

**Scheme 1:**
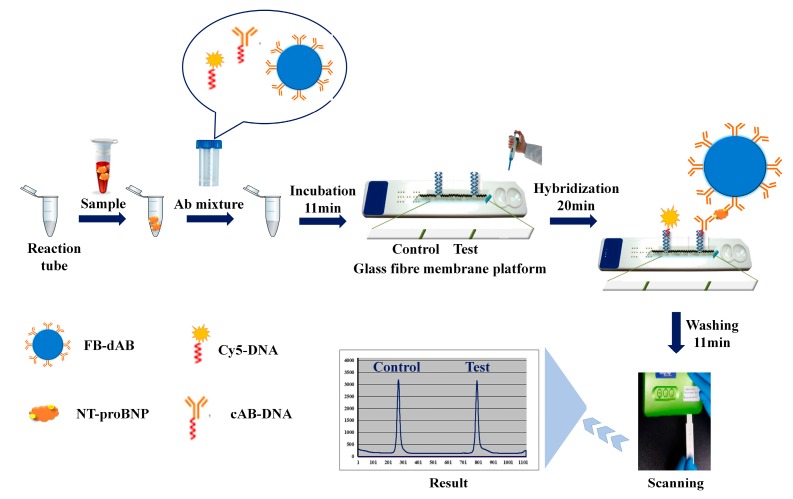
A simple and fast detection of NT-proBNP level in the clinical samples by DAGON method on the glass fibre membrane platform.

As depicted in Scheme 1, *us*-NT-proBNP test allows the quantification of NT-proBNP in clinical samples with a simple and rapid process. In brief, *us*-NT-proBNP test is performed as follows. 10 µL of a sample was transferred into a reaction tube and then 100 µL of antibody mixture (kept under thermal control at 25 °C for 10 min) containing capture antibody-DNA (cAb-DNA) conjugate, Fluorescent bead labeled detection antibody (FB-dAB) was added. This solution was incubated in homogeneous phase for 11 min. A biomolecular complex containing NT-proBNP (FB-dAB-NT-proBNP-cAb-DNA) is formed in the solution during the incubation step. After incubation, 60 µL of reaction buffer was added to the reaction tube. The whole reaction mixture is transferred to the sample loading port of the lateral flow membrane test strip (LFMTS), where biomolecular complexes are captured on the test line by DNA-DNA hybridization of immobilized DNA probes and a DNA in the FB-dAB-NT-proBNP-cAb-DNA. The Cy5-DNA hybridizes with the immobilized probes on the control line. After 20 min hybridization, the unbound biomolecular complexes and other components were removed by loading the 170 µL washing solution in the washing port of LFMTS. After the washing step, the LFMTS were scanned in the BMT Reader™ (Biometrix Technology Inc. Chuncheon, South Korea) to obtain the results. BMT Reader™ is a lightweight, portable device that measures fluorescence signals. Every step of the experiment is performed at a room temperature. NT-proBNP concentrations in the clinical samples (measurements in triplicate) were compared with the results of Elecsys^®^ NT-proBNP assay.

### 2.3. Standard Curve of us-NT-proBNP Test

A standard curve was obtained by diluting a stock solution of recombinant NT-proBNP in analyte-free human plasma (10 calibration points; 0–600 pg/mL). The concentration of a stock solution was matched by dissolving the contents of the vial as per the manufacturer’s protocol. The mean of all ten values (SD in the range of 2.1–9.5%) for fluorescence signal measurements of each calibration points were used to construct the standard curve. Effect of the change in the instrument on the measurements, which is insignificant, was determined by using eight BMT Reader™ instruments (Biometrix Technology Inc. Chuncheon, South Korea) for all data points.

### 2.4. Elecsys^®^ NT-proBNP Assay (NT-proBNP Assay)

The concentration of NT-proBNP in all samples was determined according to the manufacturer’s protocol using an Elecsys^®^ NT-proBNP assay (Roche Diagnostics GmbH, Mannheim, Germany) on a Roche Elecsys^®^ E601 analyzer (Roche Diagnostics, Changning, China).

### 2.5. Analytical Performance

The limit of blank (LoB) and limit of detection (LoD) were determined according to the Clinical and Laboratory Standards Institute (CLSI) EP17-A requirements [36]. The LoD signifies the 95th percentile value from *n* ≥ 65 experiments of blank (analyte-free) samples. The dilution linearity of the test was determined by with spiking (*n* = 3) and without spiking (*n* = 5) human plasma samples with NT-proBNP in standard stock solution. Each sample was serially diluted with the analyte-free plasma to obtain the solutions with the concentrations in the detection range. Each experiment was performed in triplicate. It is crucial that a mean (SD) recovery of the measured NT-proBNP concentrations should be 100% (20%) of the expected NT-proBNP concentration to demonstrate acceptable linearity of the test.

### 2.6. Interference Testing

The possible interference of bilirubin, intra-lipid, biotin, and hemoglobin in *us*-NT-proBNP test was evaluated by spiking the plasma samples with these interfering materials. Two samples with NT-proBNP level of 200 pg/mL and 420 pg/mL were individually spiked with bilirubin (0.2 mg/mL) and intralipid (0.2%). Two samples with NT-proBNP level of 60 pg/mL and 400 pg/mL were spiked with biotin (30 ng/mL) and hemoglobin (1 mg/mL). The spiked samples were incubated for 30 min at 25 °C. Solutions with 1/2 dilutions were made for each plasma with the corresponding baseline plasma and the original, and diluted samples were measured in duplicate.

### 2.7. Statistical Analysis

Data were analyzed using Prism (GraphPad Software Inc., La Jolla, CA, USA), Excel (Microsoft Office 2013, Microsoft, Redmond, WA, USA) and Medcalc version 17.1 (Medcalc, Ostend, Belgium).

## 3. Results

**Figure 1:**
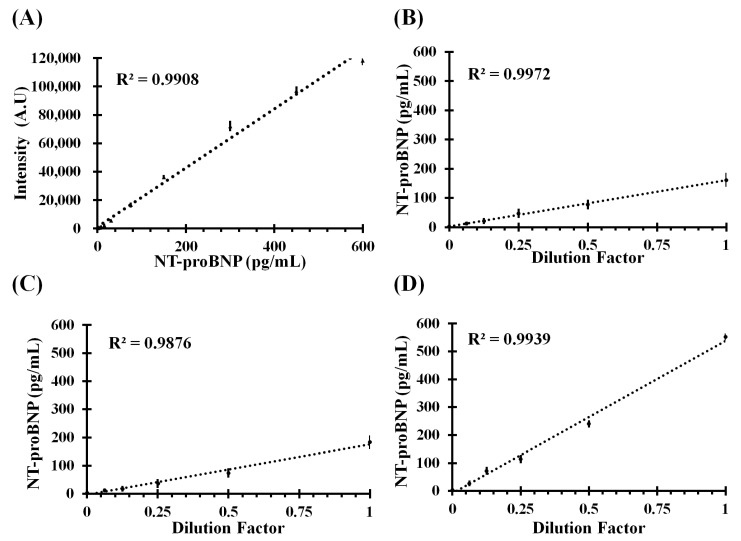
(**A**) Standard curve, (**B**–**D**) depict the linearity of the test in clinical samples containing 162 pg/mL, 183.3 pg/mL, and 551.9 pg/mL of NT-proBNP after spiking, respectively.

The LoB and LoD for *us*-NT-proBNP test were determined to be 2.0 pg/mL, 3.7 pg/mL, respectively. Linearity was documented by dilution of samples in the range of 0–600 pg/mL. Spiked samples (*n* = 3) and non-spiked samples (*n* = 5) were serially half diluted with recommended analyte-free dilution buffer until the dilution ratio of 1/16. NT-proBNP recovery (SD) was 95.1% (13.5%) in these samples. Thus, the analytical measurement range was 7–600 pg/mL. Figure 1A shows a standard curve that was constructed by using ten calibration points in the range of 0–600 pg/mL. Figure 1B–D depict the linearity of the test by spiking the human plasma samples to achieve final NT-proBNP concentrations of 162 pg/mL, 183.3 pg/mL, and 551.9 pg/mL.

As shown in Figure 1, *us*-NT-proBNP test showed a linear correlation with the concentration of the NT-proBNP in the solution. As shown in Figure 1B–D the spiked plasma samples showed a linear decrement in the concentration upon dilution. The obtained regression coefficients, which were in the range of 0.987–0.997, indicate that *us*-NT-proBNP test show linearity in the quantification of NT-proBNP in serially diluted samples. The mean (SD) of spike recovery was found to be 95.1% (13.5%). ESI Figure S1, demonstrate linearity in dilution test of five non-spiked plasma samples containing 83.8 pg/mL, 79.8 pg/mL, 65.2 pg/mL, 52.9 pg/mL, and 49.1 pg/mL of NT-proBNP.

**Figure 2:**
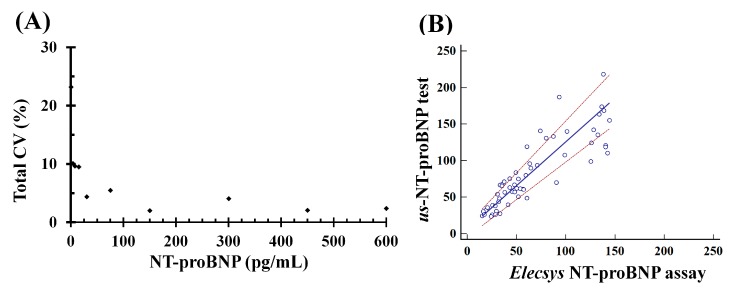
(**A**) Inter-assay precision profiles for the *us*-NT-proBNP test; (**B**) Passing and Bablok regression analyses of results for 58 clinical samples (range: 26.3–163.3 pg/mL) by *us*-NT-proBNP test and NT-proBNP assay.

According to reports, a good precision (CV < 10%) indicates the high sensitivity of the assay [37,38]. Therefore, to assess the sensitivity of *us*-NT-proBNP test, within-run precision and between-run precision was determined by using clinical and control samples. Figure 2A depicts the inter-assay precision of *us*-NT-proBNP test. An imprecision corresponding to CV< 10% was observed across the entire detection range of 7.0–600 pg/mL in tested samples. For a sample, containing 3.5 pg/mL of NT-proBNP the value of CV (14.4%) was found to be slightly higher than other samples. A correlation between *us*-NT-proBNP test and NT-proBNP assay by Passing and Bablok regression analyses of 58 clinical samples is demonstrated in the Figure 2B. The correlation coefficient between the two tests was found to be 0.899 (95%CI: 0.834–0.939; *p* < 0.0001). A slight absolute bias, indicated by the y-intercept (y = 1.2x + 5.73), was observed. Passing and Bablok regression analyses determined the proportional and random differences to be 1.2 and 16.3, respectively, indicating there was no significant deviation from linearity (*p* = 0.54). Overall, there was no significant difference between the average concentrations measured with *us*-NT-proBNP test and NT-proBNP assay on these 58 samples (*us*-NT-proBNP test median = 67.2 pg/mL (23.6–218.2) and NT-proBNP assay median = 52.0 pg/mL (15.2–144.4)).

**Figure 3:**
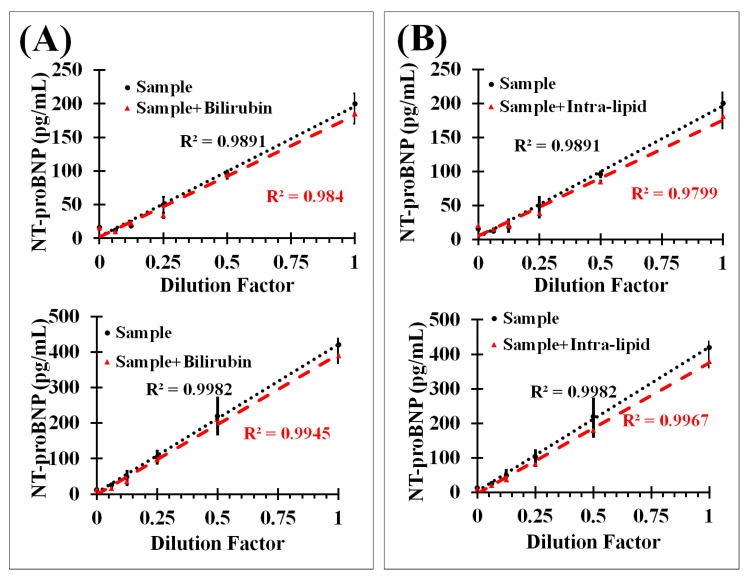
Determination of interference of (**A**) Bilirubin (0.2 mg/mL), (**B**) Intra-lipid (0.2%) emulsion (Plasma samples containing 200 pg/mL and 420 pg/mL of NT-proBNP arranged from top to bottom, respectively).

The interference from endogenous constituents such as bilirubin, intra-lipid, hemoglobin, and biotin in plasma has been reported in immunoassays used for the detection of NT-proBNP [39,40]. Therefore, to determine the interference of these materials in *us*-NT-proBNP test, plasma samples were spiked separately with bilirubin (0.2 mg/mL), intra-lipid (0.2%), hemoglobin (1 mg/mL) and biotin (30 ng/mL). Figure 3A,B demonstrate the results for samples containing 200 pg/mL and 420 pg/mL of NT-proBNP mixed with the bilirubin and intra-lipid. A serial dilution test by *us*-NT-proBNP test was also performed on the samples in presence and absence of bilirubin and intra-lipid. The similarity in the correlation coefficient values, which were higher than 0.984, in presence and absence of interfering materials clearly indicate that *us*-NT-proBNP test does not have any interference from bilirubin, intra-lipid, and any other endogenous constituents in the tested samples.

**Figure 4:**
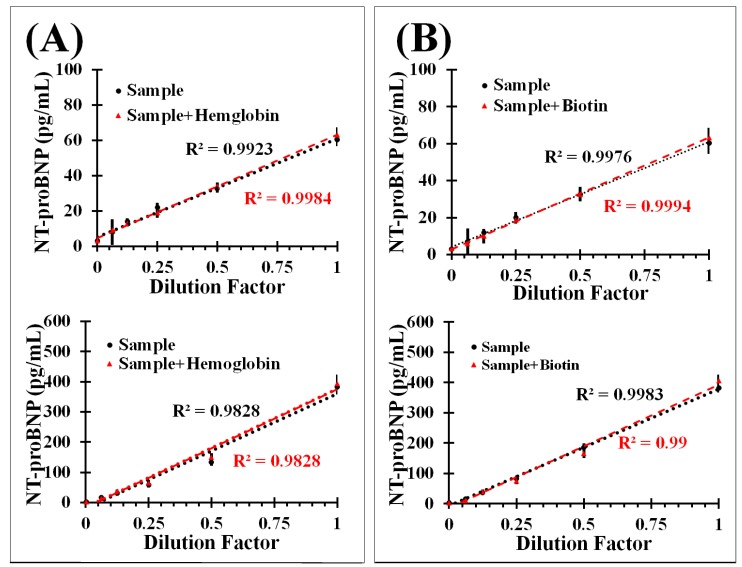
Determination of interference of (**A**) haemoglobin (1 mg/mL); (**B**) biotin (30 ng/mL) in clinical samples containing 60 pg/mL (top) and 400 pg/mL (bottom) of NT-proBNP.

Figure 4A,B demonstrates the results for samples containing 60 pg/mL and 400 pg/mL of NT-proBNP mixed with the hemoglobin and biotin. A serial dilution test of these samples in the presence and absence of hemoglobin and biotin allowed measuring the effect of these interfering materials on *us*-NT-proBNP test. The correlation coefficient values, which were higher than 0.982, indicate that hemoglobin and biotin do not have any effect on the performance of *us*-NT-proBNP test.

## 4. Discussion

The natriuretic peptides, BNP and NT-proBNP, are released from the cardiac ventricles in in response to pressure overload in left ventricles and increased stress on ventricular walls. Increased NT-proBNP levels are associated with the left ventricular dysfunction in asymptomatic individuals [41] and HF severity [42]. The elevated NT-proBNP levels are not only the indicators of structural heart disease but also relate to the incident HF in individuals within the general population [43,44].

The recent report on the prognostic value of NT-proBNP revealed that the individuals with NT-proBNP levels <19 pg/mL had all cause mortality of 0.8% which was increased to 7% in patients with the values >81.9 pg/mL. The major cardiovascular events (MACE) also showed the ten times increase with the increase in NT-proBNP levels from <19 pg/mL to >81.9 pg/mL. The study also concluded that the participants with NT-proBNP >81.7 pg/mL have a significantly higher risk of death and MACEs [20]. According to another report, the use of a cut-off point of 35 pg/mL enabled the identification LVH in participants with a sensitivity of 100% [45]. Patients with the NT-proBNP levels >125 pg/mL were found to have LVSD. Furthermore, patients who were referred for echocardiography in primary care because of suspected CHF, NT-proBNP values <125 pg/mL effectively rule out LVSD [46]. Hence, it is evident from these reports that the accurate detection of the NT-proBNP value at low concentration is highly significant for the determination of the risk factors leading to the HF.

The addition of NT-proBNP to traditional risk factors significantly improves HF risk prediction [47]. Therefore, early identification of vulnerability to HF and its efficient monitoring in general healthcare settings using NT-proBNP levels may significantly reduce the global burden of HF by enabling proactive risk management. The accurate detection of NT-proBNP levels way below the 125 pg/mL [48], which is a cutoff, can help cardiologists to determine the risk of HF, in the management of CHF and in evaluating the response to therapy in HF patients [49].

*us*-NT-proBNP test, a new method that has a detection range of 7–600 pg/mL, was developed and validated for NT-proBNP measurement in clinical samples. To validate the performance of *us*-NT-proBNP test, the precision, analytical sensitivity, and linearity were determined and the results were compared with those of the NT-proBNP assay, a reference method. It is important to note that the CV of <10% in the entire detection range indicate that *us*-NT-proBNP test demonstrates very high accuracy in the detection of NT-proBNP in clinical samples. Therefore, the specific and sensitive detection of NT-proBNP in the range of 7–600 pg/mL by *us*-NT-proBNP test promises the significant clinical advancement to reduce the mortality and costs associated with hospitalizations.

According to the reports, immunoassays suffer from the interference of plasma constituents among which bilirubin, intra-lipid, hemoglobin, and biotin are prominent [50]. The commercial immunoassays based on streptavidin-biotin interactions suffer from the interference of heterophilic antibody resulting in falsely elevated analyte concentrations [51]. The interference from an excess of biotin results in the falsely deprived analyte concentrations in the streptavidin-biotin based immunoassays [52].

Interference of endogenous substances in clinical samples result in the misinterpretation of a patient’s results leading to the wrong course of treatment [53]. It is reported that the serial dilution tests can verify the effect of interfering materials. The interference is confirmed if there is no linearity in the results [54].

The interference from endogenous materials in plasma samples on the *us*-NT-proBNP test was determined by a serial half dilution test on the selected plasma samples with and without spiking of bilirubin, intra-lipid, hemoglobin, and biotin. The *us*-NT-proBNP test showed a linear correlation in the serial dilution test of clinical samples in the presence and absences of interfering materials. The regression coefficient values higher than 0.982 indicate that *us*-NT-proBNP does not have any interference from the endogenous anti-streptavidin antibodies, autoantibodies, bilirubin, intra-lipid, hemoglobin, and biotin. The absence of interference from such endogenous plasma constituents is attributed to the highly specific DNA-DNA interactions between immobilized oligonucleotide probes and the DNAs in the DNA-capture antibody conjugate. Previous reports on the DNA-based platforms for the ultra-sensitive detection of proteins suggest that such assays do not suffer from the interference of heterophilic antibodies, autoantibodies, and biotin [55]. Therefore, *us*-NT-proBNP test is highly sensitive and highly specific with high accuracy in the detection of NT-proBNP in clinical samples.

It is known that the linearity in the serial dilution test reflects the accuracy of the measurement. The linearity in the serial dilution test is a very important characteristic of a test or an assay for its implementation in clinical practice. *us*-NT-proBNP test showed reproducible results of linearity in the serial dilution test. Hence, *us*-NT-proBNP test has high potential to implement in clinical practice.

## 5. Conclusions

The results presented herein reported indicate that *us*-NT-proBNP test showed satisfactory analytical performance for the detection of circulating NT-proBNP. The % CV was found to be less than 10% in the entire detection range of 7–600 pg/mL. The interferences from the endogenous components in the plasma have resulted in false detection of NT-proBNP at higher or lower levels in commercial assays. However, the interference test in presence and absence of bilirubin, intra-lipid, hemoglobin, and biotin demonstrated the correlation coefficients higher than 0.982. Thus, these results indicate that *us*-NT-proBNP test shows no cross-reactivity and no interference from the components of plasma samples obtained from individuals studied in this work. Overall, the results of this study indicate that *us*-NT-proBNP test is a sensitive method that offers more accurate detection of NT-proBNP values. Therefore, *us*-NT-proBNP test has a very high applicability for the detection of NT-proBNP levels in the regular health care check-up settings. *us*-NT-proBNP test can help physicians to determine the risk of HF, in evaluation of the response to therapy in HF patients, and in the management of HF.

## Supplementary Materials

The following are available online at http://www.mdpi.com/1424-8220/17/9/2116/s1, Figure S1: Linearity of test by dilution of non-spiked human plasma samples.

## References

[1] World Health Organization Cardiovascular Diseases (CVDs), Factsheet. http://www.who.int/mediacentre/factsheets/fs317/en/.

[2] Roth G.A., Forouzanfar M.H., Moran A.E., Barber R., Nguyen G., Feigin V.L., Naghavi M., Mensah G.A., Murray C.J. (2015). Demographic and epidemiologic drivers of global cardiovascular mortality. N. Engl. J. Med..

[3] World Health Organization (2011). Global Status Report on Noncommunicable Diseases.

[4] Mozaffarian D., Benjamin E.J., Go A.S., Arnett D.K., Blaha M.J., Cushman M., De Ferranti S., Després J.P., Fullerton H.J., Howard V.J. (2015). Heart disease and stroke statistics—2015 update: A Report from the American Heart Association. Circulation.

[5] Mozaffarian D., Benjamin E.J., Go A S., Arnett D.K., Blaha M.J., Cushman M., Das S.R., De Ferranti S., Després J.P., Fullerton H.J. (2016). Heart disease and stroke statistics—2016 update: A Report from the American Heart Association. Circulation.

[6] (2013). Mortality Multiple Cause Micro-Data Files. Public-Use Data File and Documentation: NHLBI Tabulations.

[7] Heidenreich P.A., Albert N.M., Allen L.A., Bluemke D.A., Butler J., Fonarow G.C., Ikonomidis J.S., Khavjou O., Konstam M.A., Maddox T.M. (2013). Forecasting the Impact of Heart Failure in the United States. Circ. Heart Fail..

[8] Hildebrandt P., Collinson P.O., Doughty R.N., Fuat A., Gaze D.C., Gustafsson F., Januzzi J., Rosenberg J., Senior R., Richards M. (2010). Age-dependent values of N-terminal pro-B-type natriuretic peptide are superior to a single cut-point for ruling out suspected systolic dysfunction in primary care. Eur. Heart J..

[9] Dickstein K., Cohen-Solal A., Filippatos G., McMurray J.J., Ponikowski P., Poole-Wilson P.A., Strömberg A., Van Veldhuisen D.J., Atar D., Hoes A.W. (2008). ESC Guidelines for the diagnosis and treatment of acute and chronic heart failure. Eur. Heart J..

[10] Kuznetsova T., Herbots L., Jin Y., Stolarz-Skrzypek K., Staessen J.A. (2010). Systolic and diastolic left ventricular dysfunction: From risk factors to overt heart failure. Expert Rev. Cardiovasc. Ther..

[11] Folsom A.R., Shah A.M., Lutsey P.L., Roetker N.S., Alonso A., Avery C.L., Miedema M.D., Konety S., Chang P.P., Solomon S.D. (2015). American Heart Association’s life’s simple 7: Avoiding heart failure and preserving cardiac structure and function. Am. J. Med..

[12] Yancy C.W., Jessup M., Bozkurt B., Butler J., Casey D.E., Drazner M.H., Fonarow G.C., Geraci S.A., Horwich T., Januzzi J.L. (2013). 2013 ACCF/AHA Guideline for the Management of Heart Failure: A Report of the American College of Cardiology Foundation/American Heart Association Task Force on Practice Guidelines. J. Am. Coll. Cardiol..

[13] Daniels L.B., Maisel A.S. (2007). Natriuretic peptides. J. Am. Coll. Cardiol..

[14] Corteville D.C.M., Bibbins-Domingo K., Wu A.H., Ali S., Schiller N.B., Whooley M.A. (2007). N-Terminal Pro—B-type natriuretic peptide as a diagnostic test for ventricular dysfunction in patients with coronary disease. Arch. Intern. Med..

[15] Mukoyama M., Nakao K., Hosoda K., Suga S., Saito Y., Ogawa Y., Shirakami G., Jougasaki M., Obata K., Yasue H. (1991). Brain natriuretic peptide as a novel cardiac hormone in humans. Evidence for an exquisite dual natriuretic peptide system, atrial natriuretic peptide and brain natriuretic peptide. J. Clin. Investig..

[16] Santaguida P.L., Don-Wauchope A.C., Oremus M.M., McKelvie R., Ali U., Hill S.A., Balion C., Booth R.A., Brown J.A., Bustamam A. (2014). BNP and NT-proBNP as prognostic markers in persons with acute decompensated heart failure: A systematic review. Heart Fail. Rev..

[17] Kara K., Lehmann N., Neumann T., Kälsch H., Möhlenkamp S., Dykun I., Broecker-Preuss M., Pundt N., Moebus S., Jöckel K.H. (2015). NT-proBNP is superior to BNP for predicting first cardiovascular events in the general population: The Heinz Nixdorf Recall Study. Int. J. Cardiol..

[18] Hildebrandt P. (2009). Natriuretic peptides: Prediction of cardiovascular disease in the general population and high risk populations. Dis. Markers.

[19] Taylor C.J., Roalfe A.K., Iles R., Hobbs F.D. (2014). The potential role of NT-proBNP in screening for and predicting prognosis in heart failure: A survival analysis. BMJ Open.

[20] Zhu Q., Xiao W., Bai Y., Ye P., Luo L., Gao P., Wu H., Bai J. (2016). The prognostic value of the plasma N-terminal pro-brain natriuretic peptide level on all-cause death and major cardiovascular events in a community-based population. Clin. Interv. Aging.

[21] Jessup M., Abraham W.T., Casey D.E., Feldman A.M., Francis G.S., Ganiats T.G., Konstam M.A., Mancini D.M., Rahko P.S., Silver M.A. (2009). 2009 Focused Update: ACCF/AHA guidelines for the diagnosis and management of heart failure in adults: A report of the American College of Cardiology Foundation/American Heart Association Task Force on Practice Guidelines. J. Am. Col. Cardiol..

[22] Ajello L., Coppola G., Corrado E., Franca E.L., Rotolo A., Assennato P. (2013). Diagnosis and treatment of asymptomatic left ventricular systolic dysfunction after myocardial infarction. ISRN Cardiol..

[23] Itoh H., Nakao K., Saito Y., Yamada T., Shirakami G., Mukoyama M., Arai H., Hosoda K., Suga S., Minamino N. (1989). Radioimmunoassay for brain natriuretic peptide (BNP) detection of BNP in canine brain. Biochem. Biophys. Res. Commun..

[24] Togashi K., Ando K., Kameya T., Kawakami M. (1991). A specific and highly sensitive radioimmunoassay of human brain natriuretic peptide. Rinsho Byori..

[25] Masson S., Vago T., Baldi G., Salio M., Angelis N.D., Nicolis E., Maggioni A.P., Latini R., Norbiato G., Bevilacqua M. (2002). Comparative measurement of N-terminal pro-brain natriuretic peptide and brain natriuretic peptide in ambulatory patients with heart failure. Clin. Chem. Lab. Med..

[26] Mainville C.A., Clark G.H., Esty K.J., Foster W.M., Hanscom J.L., Hebert K.J., Lyons H.R. (2015). Analytical validation of an immunoassay for the quantification of N-terminal pro-B-type natriuretic peptide in feline blood. J. Vet. Diagn. Investig..

[27] Lewis L.K., Raudsepp S.D., Yandle T.G., Frampton C.M., Palmer S.C., Troughton R.W., Richards A.M. (2013). Comparison of immunoassays for NTproBNP conducted on three analysis systems: Milliplex, Elecsys and RIA. Clin. Biochem..

[28] Liu Y., Wang H., Xiong C., Chai Y., Yuan R. (2017). An ultrasensitive electrochemiluminescence immunosensor for NT-proBNP based on self-catalyzed luminescence emitter coupled with PdCu@carbon nanohorn hybrid. Biosens. Bioelectron..

[29] De Ávila B.E., Escamilla-Gómez V., Campuzano S., Pedrero M., Pingarrón J.M. (2013). Disposable amperometric magnetoimmunosensor for the sensitive detection of the cardiac biomarker amino-terminal pro-B-type natriuretic peptide in human serum. Anal. Chim. Acta.

[30] Song K., Nimse S.B., Kim J., Kim J., Ta V., Nguyen V., Kim T. (2011). 9G DNAChip: A platform for the efficient detection of proteins. Chem. Commun..

[31] Sonawane M.D., Nimse S.B., Song K., Kim T. (2017). Multiplex detection of cardiac biomarkers. Anal. Methods.

[32] Song K., Nimse S.B., Kim J., Sayyed D.R., Kim T. (2013). A new platform for a convenient genotyping system. Chem. Commun..

[33] Song K., Nimse S.B., An H., Kim T. (2014). HPV Genotyping 9G membrane test: A point-of-care diagnostic platform. Sensors.

[34] Song S.Y., Han Y.D., Kim K., Yang S.S., Yoon H.C. (2011). A fluoro-microbead guiding chip for simple and quantifiable immunoassay of cardiac troponin I (cTnI). Biosens. Bioelectron..

[35] Jung Y., Lee J.M., Jung H., Chung B.H. (2007). Self-directed and self-oriented immobilization of antibody by protein G-DNA conjugate. Anal. Chem..

[36] Tholen D.W., Linnet K., Kondratovich M., Armbruster D.A., Garrett P.E., Jones R.L., Kroll M.H., Lequin R.M., Pankratz T.J., Scassellati G.A. (2004). Protocols for Determination of Limits of Detection and Limits of Quantitation; Approved Guideline.

[37] Panteghini M., Pagani F., Yeo K.T., Apple F.S., Christenson R.H., Dati F., Mair J., Ravkilde J., Wu A.H., Committee on Standardization of Markers of Cardiac Damage of the IFCC (2004). Evaluation of imprecision for cardiac troponin assays at low-range concentrations. Clin. Chem..

[38] Apple F.S., Parvin C.A., Buechler K.F., Christenson R.H., Wu A.H., Jaffe A.S. (2005). Validation of the 99th percentile cutoff independent of assay imprecision (CV) for cardiac troponin monitoring for ruling out myocardial infarction. Clin. Chem..

[39] Yeo K.T., Wu A.H., Apple F.S., Kroll M.H., Christenson R.H., Lewandrowski K.B., Sedor F.A., Butch A.W. (2003). Multicenter evaluation of the Roche NT-proBNP assay and comparison to the biosite triage BNP assay. Clin. Chim. Acta.

[40] Zugck C., Nelles M., Katus H.A., Collinson P.O., Gaze D.C., Dikkeschei B., Gurr E., Hayen W., Haass M., Hechler C. (2006). Multicentre evaluation of a new point-of-care test for the determination of NT-proBNP in whole blood. Clin. Chem. Lab. Med..

[41] Luers C., Wachter R., Kleta S., Uhlir M., Koschack J., Scherer M., Binder L., Herrmann-Lingen C., Zapf A., Kulle B. (2010). Natriuretic peptides in the detection of preclinical diastolic or systolic dysfunction. Clin. Res. Cardiol..

[42] Ndumele C.E., Matsushita K., Sang Y., Lazo M., Agarwal S.K., Nambi V., Deswal A., Blumenthal R.S., Ballantyne C.M., Coresh J. (2016). N-Terminal Pro-Brain Natriuretic Peptide and Heart Failure Risk Among Individuals With and Without Obesity: The Atherosclerosis Risk in Communities (ARIC) Study. Circulation.

[43] Wang T.J., Gona P., Larson M.G., Tofler G.H., Levy D., Newton-Cheh C., Jacques P.F., Rifai N., Selhub J., Robins S.J. (2006). Multiple biomarkers for the prediction of first major cardiovascular events and death. N. Engl. J. Med..

[44] Defilippi C.R., Christenson R.H., Gottdiener J.S., Kop W.J., Seliger S.L. (2010). Dynamic cardiovascular risk assessment in elderly people. The role of repeated N-terminal pro-B-type natriuretic peptide testing. J. Am. Coll. Cardiol..

[45] Morillas P., Castillo J., Quiles J., Nuñez D., Guillén S., Maceira A., Rivera M., Bertomeu V. (2008). Usefulness of NT-proBNP level for diagnosing left ventricular hypertrophy in hypertensive patients. A cardiac magnetic resonance study. Rev. Esp. Cardiol..

[46] Gustafsson F., Steensgaard-Hansen F., Badskjaer J., Poulsen A.H., Corell P., Hildebrandt P. (2005). Diagnostic and prognostic performance of N-terminal ProBNP in primary care patients with suspected heart failure. J. Card. Fail..

[47] Agarwal S.K., Chambless L.E., Ballantyne C.M., Astor B., Bertoni A.G., Chang P.P., Folsom A.R., He M., Hoogeveen R.C., Ni H. (2012). Prediction of incident heart failure in general practice: The Atherosclerosis Risk in Communities (ARIC) Study. Circ. Heart Fail..

[48] Ponikowski P., Voors A.A., Anker S.D., Bueno H., Cleland J.G., Coats A.J., Falk V., González-Juanatey J.R., Harjola V.P., Jankowska E.A. (2016). 2016 ESC Guidelines for the diagnosis and treatment of acute and chronic heart failure: The Task Force for the diagnosis and treatment of acute and chronic heart failure of the European Society of Cardiology (ESC)Developed with the special contribution of the Heart Failure Association (HFA) of the ESC. Eur. J. Heart Fail..

[49] Dietl A., Stark K., Zimmermann M.E., Meisinger C., Schunkert H., Birner C., Maier L.S., Peters A., Heid I.M., Luchner A. (2016). NT-proBNP Predicts Cardiovascular Death in the General Population Independent of Left Ventricular Mass and Function: Insights from a Large Population-Based Study with Long-Term Follow-Up. PLoS ONE.

[50] Januzzi J.L., Lewandrowski K.B., Bashirians G., Jackson S., Freyler D., Smith K., Murakami M.M., Apple F.S. (2008). Amino-terminal Pro—B-type natriuretic peptide testing for the diagnosis or exclusion of heart failure in patients with acute symptoms. Am. J. Cardiol..

[51] Ando T., Yasui J., Inokuchi N., Usa T., Ashizawa K., Kamihara S., Eguchi K. (2007). Non-specific activities against ruthenium crosslinker as a new cause of assay interference in an electrochemilluminescent immunoassay. Intern. Med..

[52] Vos M.J., Rondeel J.M.M., Mijnhout G.S., Endert E. (2017). Immunoassay interference caused by heterophilic antibodies interacting with biotin. Clin. Chem. Lab. Med..

[53] Saleem M., Lewis J.G., Florkowski C.M., Mulligan G.P., George P.M., Hale P. (2009). A patient with pseudo-Addison’s disease and falsely elevated thyroxine due to interference in serum cortisol and free thyroxine immunoassays by two different mechanisms. Ann. Clin. Biochem..

[54] Oostendorp M., Lentjes E.G. (2017). Utility of dilution tests in investigating interference in the free thyroxine assay. Clin. Chem. Lab. Med..

[55] Janssen K.P.F., Knez K., Spasic D., Lammertyn J. (2013). Nucleic Acids for Ultra-Sensitive Protein Detection. Sensors.

