# Supplementary material for: Ultra-Sensitive NT-proBNP Quantification for Early Detection of Risk Factors Leading to Heart Failure
Source: Sensors (Basel). 2017 Sep 14;17(9):2116. doi: 10.3390/s17092116 (PMC5620655; doi:10.3390/s17092116)
Supplement: Supplementary file 1 [file sensors-17-02116-s001.pdf]

Ultra-sensitive NT-proBNP Test for Early Detection of Risk Factors Leading to Heart Failure

Keum-Soo Song, Satish Balasaheb Nimse, Mukesh Digambar Sonawane, Shrikant Dashrat Warkad and Taisun Kim*

Institute for Applied Chemistry and Department of Chemistry, Hallym University, Chuncheon, 200-702, Korea; tskim@hallym.ac.kr

Supporting Information

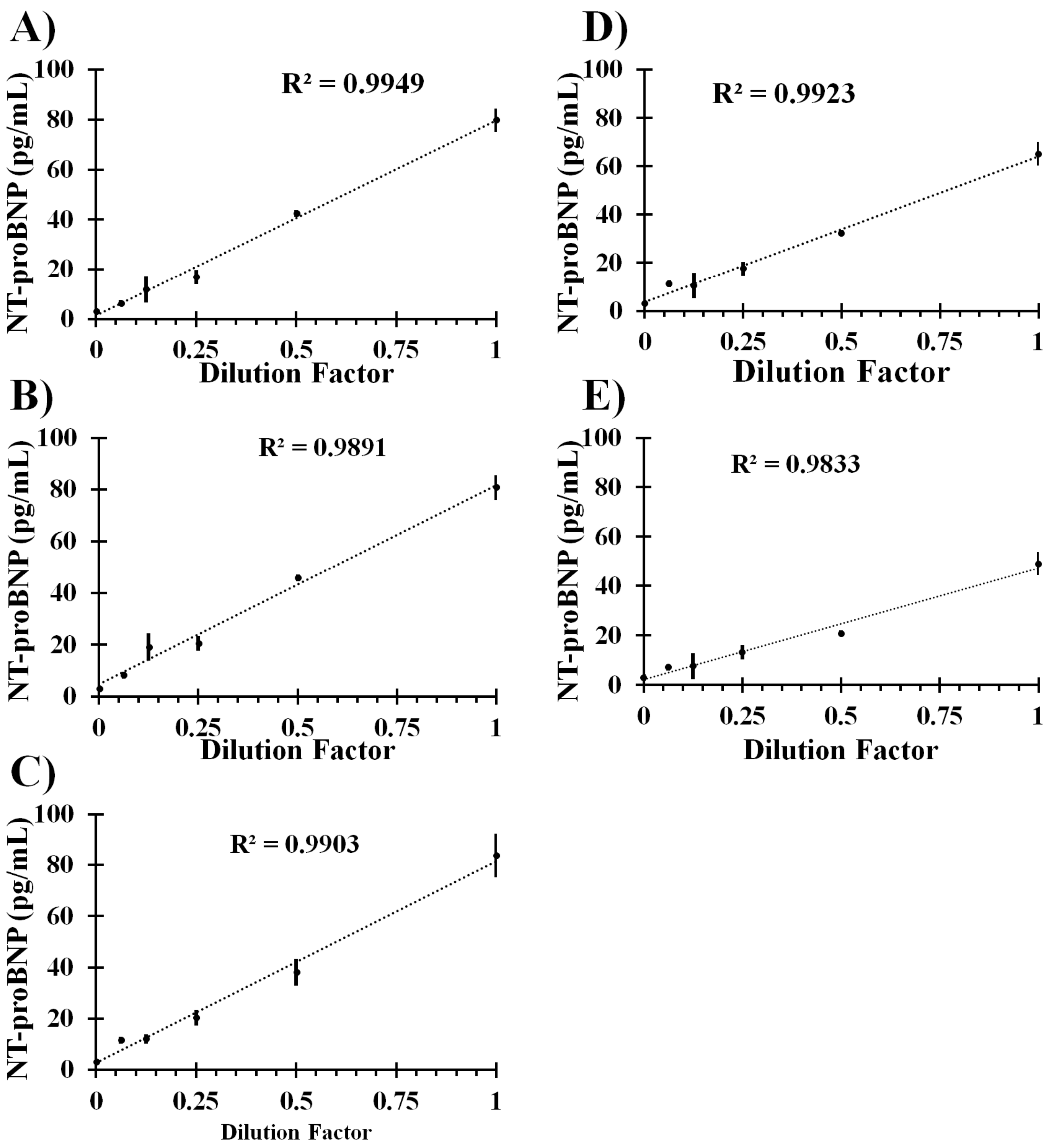

Figure S1: Linearity of test by dilution of non-spiked human serum samples containing A) 83.8pg/mL, B) 79.8 pg/mL, C) 65.2 pg/mL, D) 52.9 pg/mL, E) 49.1pg/mL NT-proBNP, respectively.
